# Let's talk about sex: older people's views on the recognition of sexuality and sexual health in the health‐care setting

**DOI:** 10.1111/hex.12418

**Published:** 2015-10-08

**Authors:** Michael Bauer, Emily Haesler, Deirdre Fetherstonhaugh

**Affiliations:** ^1^ Australian Centre for Evidence Based Aged Care (ACEBAC) La Trobe University Melbourne Vic. Australia

**Keywords:** attitudes, elderly, health‐care professionals, sexual health, sexuality, systematic review

## Abstract

**Objective:**

To report on the findings of a systematic review which examined the experiences and views of older people aged 65 years and over on health professionals’ recognition of sexuality and sexual health and whether these aspects of the person are incorporated into care.

**Review methods:**

The review followed the methods laid out by the Joanna Briggs Institute. Eleven electronic databases were searched using the terms sexual*, aged, ageing/aging, attitudes and care in any health‐care setting. Only quantitative and qualitative research and opinion papers written in English and offering unique commentary published between January 2004 and January 2015 were eligible.

**Results:**

A total of 999 papers were initially identified and of these, 148 were assessed by two reviewers. Eighteen studies – seven quantitative, eight qualitative and three opinion papers – met the inclusion criteria and were appraised. The importance of sexuality to well‐being, language used, expressing sexuality, discomfort discussing sexuality, inadequate sexuality health education and treatment and deficient communication with health‐care professionals were all identified as significant issues in a range of settings. Fourteen categories and five syntheses summarize the 43 findings.

**Conclusions:**

Sexuality remains important for many older people; however, embarrassment, dissatisfaction with treatment, negative attitudes and seeming disinterest by health professionals can all inhibit discussions. Professionals and health‐care services need to adopt strategies and demonstrate characteristics which create environments that are more supportive of sexuality. Issues related to sexuality and sexual health should be able to be discussed without anxiety or discomfort so that older people receive optimal care and treatment.

## Background

Sexuality, sexual health and the expression of sexual identity are recognized as central components of quality of life and well‐being.^1^, ^2^, ^3^ Older people are no exception, as research has consistently shown that sexuality remains important to adults over 65 years of age.^4^, ^5^, ^6^ However, the importance of sexuality for older people, including those living with dementia, is often overlooked or underestimated,^4^, ^7^ and there is a tendency to accept ageist stereotypes of older people as sexless and undesirable.^8^, ^9^

Despite the significance of sexuality to quality of life and the importance of sexual health at all life stages, much of the existing research suggests that older people's expression of sexuality is usually overlooked in health‐care settings.^7^, ^10^, ^11^ Both the literature and anecdotal evidence indicate that health‐care professionals neglect this area of care for those over the age of 65 years.^12^, ^13^, ^14^, ^15^ Surveys suggest professionals from all health‐care specialties and clinical settings have a lack of knowledge of sexuality with regard to older people and fail to adequately address their sexual health.^16^, ^17^, ^18^, ^19^ When it comes to sexuality and older people, it appears that many health‐care professionals harbour negative attitudes and exhibit behaviour which does not enable the discussion of this topic with the older person.^4^

Research suggests that offering health‐care professionals education and more exposure to older people, including those who are non‐heterosexual, may lead to a change in knowledge and attitudes;^20^ however, the provision of appropriate education that addresses older people's sexuality requires a clear understanding of their needs and preferences. This perspective is sparsely represented in the literature. This study aims to address this gap in understanding and reports on a systematic review which examines older people's perspectives on the recognition of and attitudes towards sexuality and sexual health in people aged 65 and over by health‐care professionals.

## Review methods

### Search strategy

A systematic search of the literature was conducted in MEDLINE, CINAHL, ProQuest, Google Scholar, EMBASE, Cochrane library, Web Science, Science Direct, Ageline, CABI and J‐GATE using combinations of the key search terms: sexual*, aged, ageing/aging, attitudes and care. Studies concerning people aged 65 years and over in any health‐care setting (e.g. hospital, general practice, residential aged care and community care) written in English were considered for inclusion. The outcome measure was older people's opinions on attitudes or practices associated with recognition, inclusion or exclusion of sexuality by health‐care professionals as a consideration in care they provide.

Our initial search included papers published after 1989; however, review of many of the earlier references indicated extensively dated material. We therefore limited the review to studies published between January 2004 and February 2015. Quantitative and qualitative research and opinion papers offering unique commentary (that is, information that did not emerge in research studies) were eligible. Discussion literature and systematic reviews, news articles and conference abstracts were excluded.

### Data extraction and analysis

All articles meeting inclusion criteria based on title/abstract were critically appraised by two independent reviewers, and data were extracted using the suite of standardized data extraction tools for different study designs developed by the Joanna Briggs Institute (JBI).^22^ The JBI appraisal system assigns a default quality of high to RCTs and pseudo‐RCTs and low to descriptive and case studies, with studies downgraded based on the risk of bias.^21^ For randomized controlled trials (RCTs) and pseudo‐RCTs, appraisal considers randomization, blinding, allocation concealment, description of withdrawals, comparability on entry, equivalent treatment besides the intervention of interest, reliable outcome measurement and appropriate statistical analysis.^22^ For descriptive and case series research, randomization, sample inclusion criteria, reporting of confounding factors, objective and reliable outcome measurement, appropriate comparative analysis and description of withdrawals are appraised.^22^

The JBI appraisal system considers qualitative research to provide a default level of high dependability. Dependability of qualitative evidence may be downgraded when there is incongruity between the research methodology and the research question, objectives, data collection methods or data analysis techniques, when reflexivity is lacking, or when the conclusion does not logically flow from the data. Text and opinion resources provide a default level of low dependability and may be downgraded when the source of opinion has no standing or is unsupported by peers, when the argument is not analytical, or when any incongruence with extant literature is not addressed.^21^

### Data analysis

Qualitative studies were analysed according to methods described by JBI^22^ to identify themes, concepts and meanings within the research. Primary findings were identified and direct quotes from the texts were compiled to illustrate these findings. Primary findings were grouped into categories based on similarity in meaning and then meta‐aggregated. The outcome measures in the quantitative research were not appropriate for meta‐analysis, and studies are reported in a narrative format within the qualitative synthesis to which they relate.

## Identified research

**Figure 1:**
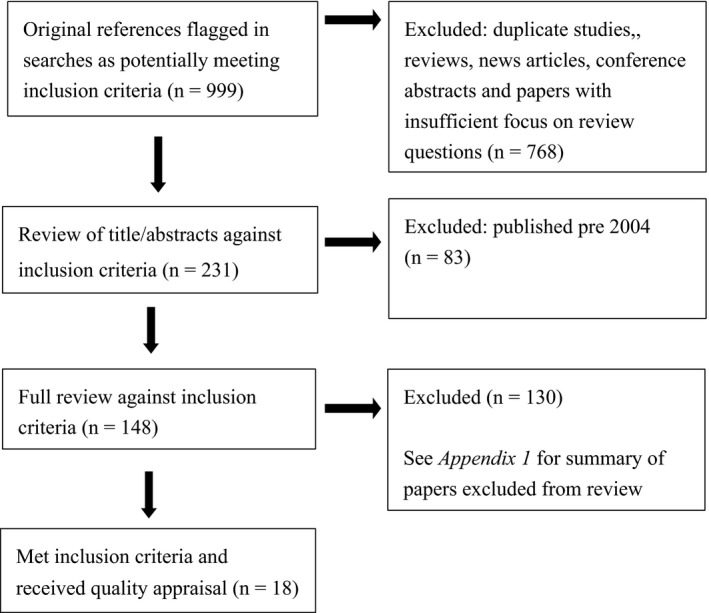
PRISMA review flow.

The Preferred Reporting Items for Systematic Reviews and Meta‐Analyses (PRISMA) flow diagram^23^ is presented in Fig. 1. The initial searches identified 999 studies that potentially addressed the area of interest. After initial review of title/abstract, 231 studies were flagged, and this was reduced to 148 after exclusion of papers published before 2004. Following a full review of these papers, 130 were rejected as they did not meet the review objective or the inclusion criteria (see Table S3). Methodological appraisal was conducted on the 18 papers identified for inclusion.

The 18 papers included in this review were heterogeneous in their methods, focus and settings and represented older adults in oncology care, cardiology care, medical centres, general practice, nursing homes, assisted living and the community. Seven papers used quantitative methods, six of which were low or very low quality^24^, ^25^, ^26^, ^27^, ^28^, ^29^ and one of moderate quality.^30^ Of these seven studies, six^24^, ^25^, ^26^, ^27^, ^28^, ^29^ were cross‐sectional survey designs using primarily non‐validated tools and were subject to response bias. The seventh study was an RCT^30^ comparing the effectiveness of different questioning styles in eliciting sexual health information. Qualitative sources consisted of eight research studies and three opinion papers containing unique data. Three qualitative studies were of high dependability,^4^, ^5^, ^31^ one moderate dependability^30^ and four were of low dependability.^32^, ^33^, ^34^, ^35^ The opinion papers were all of low dependability.^36^, ^37^, ^38^ The qualitative studies were underpinned by a range of philosophical perspectives and used in‐depth interviewing or focus groups to collect data (see Tables S1 and S2 for summaries of the included papers).

## Review findings

**Figure 2:**
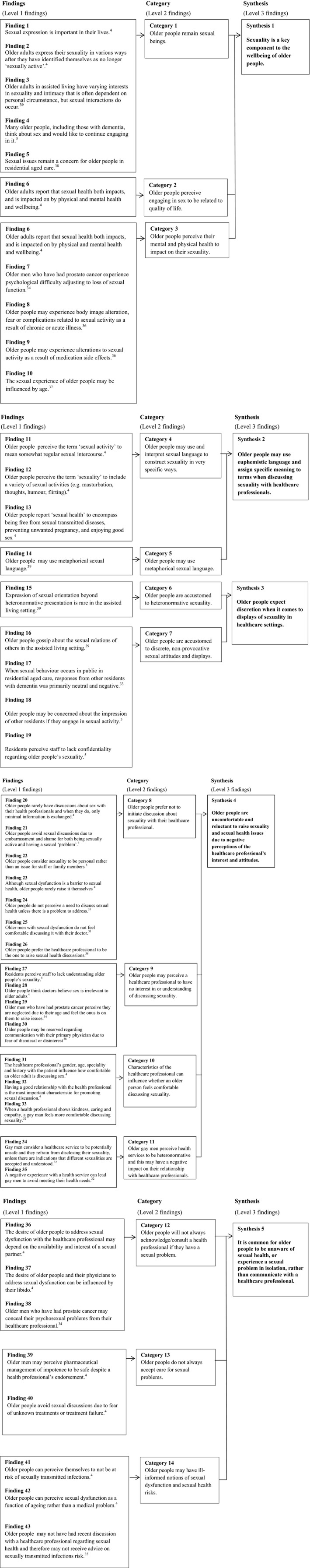
Synthesis of the findings about older people's views on sexuality.

Forty‐three findings were extracted from the included studies and grouped into 14 categories. Five syntheses summarized both quantitative and qualitative evidence and broadly addressed perceptions of the importance of sexuality to the well‐being of older people, the language they use when discussing sexuality, issues related to displaying sexuality in the health‐care setting, the discomfort that older people can feel in raising sexual issues with their health‐care provider, and the paucity of sexual health education and treatment when communication about sexuality is poor (see Fig. 2).

Synthesis 1: Sexuality is a key component to the well‐being of older people.

The first synthesis relates to the importance of sexuality to older people's quality of life. Older people acknowledged that their physical and mental health impacts on their sexual health.^4^ Age‐related factors such as declining fitness and mobility levels, hormonal changes and specific diseases (e.g. arthritis)^37^ as well as psychological factors^36^, ^37^ including loss of self‐esteem, decreased confidence in performance, the potential for a decline in cognitive function and medication side‐effects,^24^, ^36^, ^37^ can all impact on older people's sexuality. In one cross‐sectional survey, people aged over 65 years with heart failure were significantly more likely (19 vs. 1%, *P* < 0.001) to experience shortness of breath that impacted on their sexual function.^26^ In another study, older men who had experienced prostate cancer reported difficulty adjusting to the impact of this illness on their sexual function:

At first I felt in a sense that I was letting my wife down … I was hoping to have a satisfying sexual relationship with her … I did feel that I was not fully a man not being able to sort of function 100%.

(age 79; hormone therapy),^34^ p. 204

Despite the negative influence of physical and psychological ill‐health and the consequences of normal ageing, the research is clear that older people continue to be sexually active and receive physical and psychological benefit from this.^4^, ^5^, ^38^, ^39^ Hoekstra, Lesman‐Leegte^26^ reported that although older people with heart failure rated sex as being less important, there was no significant difference in their overall interest in sex compared with healthy older adult controls. In a large cohort of women attending a medical centre for a Pap smear (*n* = 1480), Nusbaum *et al*.^28^ found no difference in sexual interest levels between older (aged over 64 years) and younger women. Bauer *et al*.^5^ found older people in residential care both with and without dementia, still considered sexual intimacy to be important:

Oh yes, yes, yes, I do miss intimacy … companionship and love.

(87 year old man with dementia),^5^ p. 301

The research also suggests that older people's interest in sexuality is not confined to just reminiscing and desires. Participants (*n* = 279) in ethnographic studies conducted in assisted living facilities in the United States (*n* = 13), confirmed that older people frequently also engaged in sexual activity after relocating to residential aged care.^39^

Synthesis 2: Older adults may use euphemistic language and assign specific meaning to terms when discussing sexuality with health‐care professionals.

The second synthesis addresses the ways in which older people refer to sexual activity and sexual health. In a grounded theory study^4^ conducted in community settings in the United States, older people (*n* = 25) interpreted sexual terms very specifically. The term ‘sexual activity’ was considered to refer to sexual intercourse only, whereas the term ‘sexuality’ was interpreted more broadly to include sexual desire and libido, humour and flirtation, sexual thoughts and masturbation. Sexual health was understood to mean being free from sexually transmitted diseases and unwanted pregnancy, as well as enjoying a good quantity and quality of sex.^4^ Use of metaphorical language as well as euphemism was noted:

Snow on the mountain, fire in the furnace – just because I'm old don't mean the other parts of me aren't hot.

(older woman in assisted living),^39^ p. 30

Synthesis 3: Older people expect discretion when it comes to displays of sexuality in health‐care settings.

Expectations of the ways in which sexuality can be acceptably expressed have been noted in the research conducted in aged care facilities. Frankowski and Clark^39^ found that the expression of sexual orientation outside the heteronormative was rare in US‐assisted living facilities. When non‐heterosexuality was observed, it often elicited a negative response from other older residents.

However, heterosexual older people living in nursing home settings can also face negative attitudes and gossip from both other residents and health‐care staff if intimate relationships or other sexual expressions are observed or suspected.^5^, ^39^ Older people considered privacy and discretion to be paramount, both for themselves^5^ and with respect to the sexual behaviours exhibited by other residents.^33^, ^39^ Where environments are not discrete, many older people may refrain from sexual activity:

… everybody would know about it and they'd be ‘yap yap yap’.

(85 year old man with dementia),^5^ p. 304

Tzeng *et al*.^33^ noted that residents with dementia are an exception when it comes to discretion as they may lack inhibitory control. Overt displays of sexuality through public masturbation, inappropriate approaches towards others and other sexual indiscretion, can be an unfortunate consequence of cognitive impairment. In Tzeng *et al*.'s grounded theory research that reported observations of, and interviews with, older Taiwanese people with dementia living in dementia‐specific facilities, indiscrete sexual activity was observed. Residents with dementia were also noted to respond negatively and often with distress,^33^ as the following report indicates:

Few residents displayed a significant negative response when they saw sexual behaviours…but would say words like ‘shameful’ or ‘embarrassing’ to the perpetrator…a female resident with dementia observer became emotional and shouted ‘Where are the nurses?’

33, p. 997

Synthesis 4: Older people are uncomfortable and reluctant to raise sexuality and sexual health issues due to negative perceptions of the health‐care professional's interest and attitudes.

The research indicates that older people often feel uncomfortable or reluctant to discuss sexuality with health‐care professionals, particularly if they have to raise issues of their own accord. Quantitative evidence highlights that older women in particular are reluctant to initiate a discussion about sexual health. In a survey completed by 101 older adults in retirement and community‐based services, Farrell and Belza^24^ found that the majority of respondents would feel too embarrassed to discuss sexuality, despite approximately 40% wanting to be asked about their sexual health. In this study, men were significantly more likely than women to both have a question about sexual health (41.7 vs. 6.2%, *P* < 0.01) and to want a discussion with the health‐care professional about it (72 vs. 37.1%, *P* < 0.01). However, almost 45% of participants placed the onus on the health‐care professional to initiate a discussion.^24^ In a cohort of women receiving care at a US military centre (*n* = 1450), women aged over 65 years were significantly less likely than younger women to have ever had a discussion with their health‐care professional about a sexual issue (33 vs. 52%, *P* < 0.001).^28^ In the RCT conducted by Sadovsky *et al*.^30^ 22% of women who were sexually active had a desire to discuss sexual health with their clinician, but had not raised their sexual problems in consultations.

In interviews with 25 older people, Colton^4^ established that the participants rarely had discussions about sexuality with their health‐care professionals, and when they did, minimal information was exchanged. Bauer *et al*.^5^ found that residents in nursing homes avoided discussions with health‐care professionals because they felt their sexuality was a personal matter and not an issue for staff members. Studies have found that conversations were rarely initiated by the older person due to the shame, embarrassment and discomfort they felt from being a sexually active being with a sexual health problem.^4^, ^31^, ^35^, ^38^

Yeah, like I…right now I'm pushing eighty. I think I'd be a weirdo [to bring sex up with the doctor].

4, p. 67

The perception that health‐care staff are uninterested in, or lack understanding of older people's sexuality also prevent older people from initiating discussions about their sexuality. Residents in Australian nursing homes expressed a perception that facility staff had a lack of understanding about their sexuality^5^ and, in another study, men with prostate cancer who had sexual concerns felt they were neglected by health‐care professionals due to their age.^34^ Fear of being dismissed, or the health‐care professional simply being uninterested in their needs, was a common issue raised in the qualitative research evidence.^4^, ^34^, ^38^

They [doctors] look at the white hair and stopped asking.

35, p. 345

Perception of the health‐care professional's attitude to sexuality was of particular relevance to gay men, who were more likely to refrain from disclosing their sexuality if they felt the health service was unsafe, particularly if they had experienced negativity in the past.^32^ Although Mostade^27^ reported that over two‐thirds (69.5%) of older gay men in a community setting in the United States had disclosed their sexuality to their physician, comfort in one's non‐heteronormativity was a contributing factor to disclosure. Clover^32^ found gay older men in the UK looked for indications that the health‐care professional was accepting and understanding before discussing their sexual orientation or problems.

I would like to know in advance that it was OK to say that I'm gay…not to have to learn afterwards.

32, p. 46

The personal characteristics of the health‐care professional were identified as significant in influencing the older person to discuss sexuality. In the study by Nusbaum *et al*.^28^ conducted in older women receiving a Pap smear (*n* = 1480), respondents identified a physician's characteristics that made them feel comfortable discussing sexual issues. Seeming concerned (99%), appearing comfortable with the topic (98%), being kind and understanding (98%), having a professional demeanour (96%) and having seen the health‐care professional in the past (92%) were all considered important characteristics. Women were less likely to be open about sexuality on the other hand if the physician appeared rushed (52%), impersonal (41%), not concerned (32%) or embarrassed (31%). Older people interviewed by Colton^4^ concurred that having a good relationship with the health‐care professional was the most important factor in promoting sexual discussion and gay men identified the health professional's empathetic characteristics as promoting their own comfort in disclosing their sexuality.^32^ Interviewees also identified the health‐care professional's gender, age, speciality and history with the patient, as factors that contributed to a comfortable environment for the discussion of sexuality.

Certainly there are many men…would feel very, very more than uncomfortable speaking about sex to a lady. Myself included.

4, p. 62

One RCT compared two questioning techniques used by health‐care professionals to elicit sexual information during a consultation. Sexually active, non‐Caucasian women attending US medical clinics were randomized to be questioned using a direct style in which they were asked ‘do you have any sexual problems?’ (*n* = 8 older women), or a ubiquitous question in which the health professional stated ‘many women at your age/with your medical problem report problems with sex. Are you having any problems?’ (*n* = 12 older women). Significantly, more women reported sexual problems when ubiquitous questioning was used (75 vs. 12.5%, *P* < 0.05).^30^

Synthesis 5: It is common for older people to be unaware of sexual health, or experience a sexual problem in isolation, rather than communicate with a health‐care professional.

The research suggests that older people of both genders do not always consult a health professional when they have a sexual problem. Although some quantitative research indicated that having an existing sexual problem appears to increase desire to discuss issues with a clinician,^29^, ^30^ in two qualitative studies, older men with erectile dysfunction talked about concealing their issues from the health‐care professional due to discomfort discussing their sexual concerns.^31^, ^34^ A cross‐sectional study conducted in women receiving cancer treatment found that those aged over 65 years were less likely than younger women to be interested in receiving care from health professionals for sexual issues and less willing to be contacted about formal programmes to address sexual issues.^25^

Factors such as having a partner interested in engaging in sexual activity, or having an active libido, also influenced whether an older person would consult the health professional about a sexual concern.^4^ Additionally, some older men expressed frustration at or distrust of available treatments for sexual dysfunction (particularly pharmaceuticals) and resigned themselves to their loss of function, rather than communicating concerns with their physician.^4^, ^31^

I haven't spoken to the doctor again about it. I just accepted it. He would probably try you on something else so why bother.

31, p. 898

Poor communication with health‐care professionals not only led to poor management of treatment and older people experiencing sexual difficulties in isolation, but it also contributed to lower levels of counselling and education about sexual risk, specifically sexually transmitted infections (STIs). In one cross‐sectional study (*n* = 101) conducted in retirement and community‐based services, 90% of respondents had never received information about HIV/AIDS and 80% had not received education about STIs. Because many sexually active older people had not had recent discussions with health‐care professionals about sexuality, they incorrectly assumed they were not at risk of STIs.^4^, ^35^

What did your provider do that helped you learn about sexually transmitted diseases? The consensus between both groups was ‘nothing’.

35, p. 345

## Discussion

This review examines an important yet neglected area of health care, namely the sexuality and sexual health of people aged 65 years and older and how health‐care professionals respond to this in practice.

The research addressing the older person's perspective and experience of sexuality in health‐care settings confirms previous literature that sexuality remains important to an older person's well‐being.^1^, ^2^, ^3^ Older people participating in the research studies included in this review identified benefits they attain from being sexually active, even while experiencing the effects of normal ageing or illness. The challenge for health‐care professionals is to acknowledge and uphold the importance of sexuality in older age including in nursing home settings. This can be performed by enabling open discussions about sexuality with older people and by incorporating an appreciation and understanding of sexuality into treatment and care planning. Health‐care professionals furthermore need to facilitate the creation of long‐term care environments that provide discrete sexual opportunities for older people for whom this is important.

This review has highlighted the importance of health professionals’ strong and effective communication skills, and the need for the health‐care professional to appear interested, understanding, concerned and empathetic.^4^, ^5^, ^31^, ^32^, ^34^ The evidence is clear, however, that communication between older people and health‐care professionals about sexual issues is currently poor. Although some older people express a desire for privacy,^5^ it is also evident that the older person may wish to consult their physician or other health‐care professional about sexual concerns. However, for a variety of reasons, many older people feel unable to initiate a discussion about sexuality. Older people may also need to be provided an opportunity to discuss issues related to sexuality. Health‐care professionals need to have an awareness of this possibility and adopt the essential role of arbiter and be more prepared to initiate such conversations with older people.^38^ This requires health‐care professionals to reflect on how comfortable they are discussing sexuality and their preparedness to raise this topic in the course of con‐sultations and care provision with older people.

Research has shown that education about older people and sexuality can change attitudes and improve knowledge,^17^ but further work needs to be undertaken on developing and trialling communication strategies to upskill health‐care professionals so that they are able to have discussions with older people about sexuality and sexual health. The open questioning style trialled by Sadovsky *et al*.,^30^ which normalized sexual concerns through a ubiquitous and open approach to sexual issues, could be adopted as a useful opening to discussions. This style could easily be adapted to address sexual issues in older people more broadly and to open discussions about safe sexual practices and the risk of STIs.

Developing measures that promote on‐going relationships between older people and the primary health‐care professional can be an important first step to facilitate trust and open the door to communication. In addition, being careful to define the meaning of specific terminology and clarifying what an older person may specifically mean when they use broad terms or metaphoric language will prevent misunderstanding or incorrect interpretation.

The importance of a health or care service as a safe place to expose one's apprehensions and be able to discuss sexuality has been raised by older gay men in one study included in this review. It is important for healthcare professionals to develop those characteristics that promote feelings of safety in their clients including an empathetic nature. It is also essential that a service clearly demonstrates its acceptance of all sexual orientations in service information and advertising.^32^ This strategy could be used more broadly to promote a feeling of safety for all older adults. The display and availability of posters and pamphlets in a service that portray older people in intimate and sexualized roles could be one approach to convey an attitude of openness and normalize older age sexuality. The incorporation of information about sexuality and sexual health into information booklets, promotional flyers and other documents produced for current and prospective residents of aged care facilities would also be a step towards normalizing the expression of sexuality in care environments.

## Review limitations

Although the views captured in this review are from men and women from a range of community and residential care settings, the conclusions which can be drawn are limited by a number of factors. The quality and dependability of studies was variable, and the voluntary nature of research participation in the included qualitative and cross‐sectional designs leaves results open to sampling bias. With the exception of one study conducted in Taiwan,^33^ the research included was also conducted in the USA, Europe and Australia and may therefore largely reflect a Western view of sexuality. While participants in a number of studies were noted to be culturally heterogeneous, cultural background, education and religiosity are known to influence views about sexuality^40^ and findings may not be representative of all older people.

## Conclusion

The evidence presented in this review indicates that older people consider their sexuality and its expression to be a significant component for a good quality of life. While some older people prefer their sexuality to remain in the personal realm, it is evident that there is a clear desire for many to be able to discuss sexual dysfunction or other issue related to sexuality with a health‐care professional. Negative attitudes, shame, embarrassment and a feeling that the health‐care professional is disinterested or has no treatment to offer, however, can all inhibit discussion. Strategies that promote a safe environment for sexual discussions should be implemented by health‐care services and the professionals who work there to promote open sharing of information and ensure older people receive the care, treatment, information, education and support they require. This review also highlights a need to undertake further research with older people about how they would like to be engaged in discussions about their sexuality and sexual health and how information obtained from these discussions can be incorporated into their health care and how that health care is provided.

## Funding

This project was funded using Australian Centre for Evidence Based Aged Care internal revenue.

## Conflict of interest

No conflict of interest has been declared by the authors.

## Supporting information


**Table S1.** Summary of included quantitative research.
**Table S2.** Summary of included qualitative research.

**Table S3.** Excluded papers.
