# Supplementary material for: Let's talk about sex: older people's views on the recognition of sexuality and sexual health in the health‐care setting
Source: Health Expect. 2015 Oct 8;19(6):1237–50. doi: 10.1111/hex.12418 (PMC6456814; doi:10.1111/hex.12418)
Supplement: Supplementary file 1 — Table S1. Summary of included quantitative research. Table S2. Summary of included qualitative research. [file HEX-19-1237-s001.docx]

***Table 1: Summary of quantitative research***

| **Paper** | **Aim** | **Design** | **Setting** | **Participants** | **Major findings related to the perspectives on and experiences of sexuality for older adults** |  |
| --- | --- | --- | --- | --- | --- | --- |
| ([Farrell & Belza, 2012](#_ENREF_4) ) | To measure attitudes of older adults to sexual health care | Cross sectional survey  Response rate 62% | Retirement home and community based recreation programs in Washington, US | Older adults (n=101)  73% female | Interaction with healthcare service  47.1% respondents wanted to be asked about sexual health  44.7% placed the onus on the HCP to start discussion  Most respondents would not be uncomfortable or embarrassed discussing sexuality with HCP  Attitudes to healthcare professionals  52.7% preferred a HCP of the same gender  90% had never received information HIV/AIDs  80.2% had not received information on STIs  Men were more likely than women to:   - want a discussion (72% vs 37.1%, p<0.01) - have a sexual health question (41.7% vs 6.2%) - be taking a medication that would influence their sexual health (33.3 vs 6%)   Attitudes to sexuality  Men were more likely than women to be sexually satisfied (56.5% vs 87.3%) |  |
| ([Hill et al., 2011](#_ENREF_7)) | To investigate  interest in and willingness to seek care for sexual issues | Cross sectional survey  Response rate 47.9% | Gynaecology and breast cancer centre in the US | Women aged over 18 years with cancer diagnosis (n=261)  22.6% (n=59) aged over 65 years  43.3% >12 months since last cancer treatment, 32.6% still in treatment | Interaction with healthcare service  Women aged 18 to 47 yrs more likely to be interested in receiving care for a sexual issue than women over age 65 years (odds ratio [OR] 2.94, 95% CI 1.14 to 7.54)  Women aged 48 to 67 yrs more likely to be interested in receiving care for a sexual issue than women over age 65 years (OR 4.67, 95% CI 1.72 to 12.63) Women aged 18 to 47 yrs were more likely to be willing to be contacted about a formal program to address sexual issues (aged 18-47 yrs OR 4.67, 95% CI 1.72 to 12.63)  Women aged 48 to 67 yrs were more likely to be willing to be contacted about a formal program to address sexual issues ( OR 5.40, 95% CI 1.71 to 17.04) No significant effect for marital status, type of cancer or cultural background |  |
| ([Hoekstra, Lesman-Leegte, Luttik, & et al., 2012](#_ENREF_9)) | To investigate sexual function of people with heart failure (HF) compared to healthy controls | Cross sectional survey using a tool that included a visual analogue scale (VAS) for importance and satisfaction with sex.  Response rate 59% in HF patients | Patients in a trial for management of HF and healthy community controls in the Netherlands | Patients with heart failure:  mean age 68 ± 12 years, 62% male, 68% had a current partner, 74% prescribed a beta blocker  Healthy controls:  mean age 64 ± 7 years, no health issues, 67% men, 87% had a current partner | Sexual health/activity  Adults > 65 years with HF had no significant difference compared to healthy controls of same age for interest in sex, sexual problems, arousal, erectile dysfunction, problems related to orgasm.  Adults > 65 years with HF more likely to report shortness of breath contributing to sexual function than health controls of same age (19% vs 1%, p<0.001).  Predictive factors for have a sexual problem in people > 65 years and with HF (multivariate analysis):   - being male (OR 5.18, 95% CI 3 to 8.96, p<0.001) - beta blocker (OR 2.60, 95% CI 1.43 to 4.73, p=0.002)   Attitudes to sexuality  Adults > 65 years with HF rated importance of sex to be significantly lower (p<0.05) and satisfaction to be lower (p<0.05) than healthy adults of the same age. |  |
| ([Mostade, 2004](#_ENREF_11)) | To investigate whether gay men disclose orientation to HCPs | Cross sectional using two dichotomous surveys and a validated tool called Reactions to Homosexuality Scale (RHS) | Community setting in US | Homosexual men aged over 64 years (n=105)  37.1% aged over 75 years, primarily Caucasian, fully retired, living alone, not currently partnered and primarily without any current health problems | Interaction with healthcare service  69.5% of men had disclosed sexual orientation to physician, no difference based on age (65 to74 versus >74)  Men who had self-disclosed sexual orientation (69.5%) were more likely to have completed an Enduring Power of Attorney for Health Care (p<0.01)  Men who disclosed their sexual orientation displayed less discomfort with being gay as measured by the RHS scale than those who did not disclose their sexual orientation |  |
| ([Nusbaum, Singh, & Pyles, 2004](#_ENREF_12)) | To compare sexual health concerns and their management between younger and older women | Cross sectional survey  Response rate 65.1% | US military medical centre | Women aged over 18 years attending for a Pap smear (n=1480)  17% aged over 64 years  Older women significantly more likely to be widowed and White/non-Hispanic than younger women | Sexual health/activity  No difference in mean number of sexual concerns between older women and younger (13 versus 12)  No difference in reporting of sexual aversion, interest level, having different desire to partner, unmet sexual needs or having an affair.  Older women were less likely to report having body image concerns or concerns associated with abuse.  Interaction with healthcare service  Older women were less likely than younger women to have ever had a sexual issue discussed in a consult (33% vs 52%, p<0.001)  Attitudes to healthcare professionals  Physician characteristics that promote the comfort of older women comfort in discussing sexual issues:   - physician seems concerned (99%) - comfortable with topic (98%) - kind and understanding (98%) physician has a professional demeanour (96%) - have seen physician before(92%) - physician knows them (90%)   Physician characteristics that are barriers to older women discussing sexual issues:   - physician appearing rushed (52%) - too embarrassed (58%) - impersonal physician (41%) - embarrassed physician (31%) - physician not concerned (32%) |  |
| ([Sadovsky et al., 2006](#_ENREF_15)) | To compare effectiveness of two questioning styles in eliciting information on sexual problems  Questioning style: Group 1: direct question - 'do you have any sexual problem?” (n=8 older women) Group 2: ubiquitous question - 'many women at your age/with your medical problem report problems with sex. Are you having any problems?' (n=12 older women) | RCT | Two family medical centres in urban US | Women attending clinics who were aged > 39 years, non-Caucasian and speak English were invited to participate (n=243; n=212 completed study)  n=95 were aged > 59 years and of interest to this review  Only sexually active women were randomised (n=107, n=20 aged over 59 years) | Sexual health/activity  5.8% of females >59 years responded that they were not sexually active. 86.8% of females >69 years were not sexually active.  Of women aged 61to 80 years who were sexually active (n=37):   - 24.3% had at least one sexual problem - 8.1% had pain - 8.1% had no excitation - 5.4% had no desire - 2.7% had no orgasm - 22.2% wanted to discuss the problem with their clinician   Interaction with healthcare service  For sexually active non-Caucasian women aged 61 to 80 years, significantly more reported having a sexual problem when asked the ubiquitous question compared with the direct question (75% vs 12.5%, p<0.05). |  |
| ([Smith, Honore Goltz, Ahn, Dickerson, & Ory, 2012](#_ENREF_17)) | To explore the experience of older men in discussing sexuality with physician | Cross sectional survey using and discussing sexual issues with a physician | Community dwelling in US | CVD and diabetes older men - a subset of 1,100 males aged 57 to 85 years from a national longitudinal survey, 25% aged over 69 years, 77.4% married, 75.4% white non-Hispanic, 58% had education beyond high school, 40% never diagnosed with CVD or diabetes, 43% CVD only, 17% diabetes only | Sexual health/activity  Factors associated with being more likely to discuss sex with physician:  greater than high school education OR =1.68 (95% CI=1.23 to 2.28, p=0.01; having more chronic medical conditions OR 1.62, 95% CI 1.35 to 1.96, p<0.001; having had sex in previous 12 months OR 1.73, 95% CI 1.17 to 2.57, p=0.006; trouble getting/maintaining erection OR 2.26, 95% CI 1.51 to 3.38, p<0.001 Factors associated with being less likely to discuss sex with physician: diagnosed with CVD only (not diabetes) OR 0.54, 95% CI 0.36 to 0.82, p=0.004 |  |

***Table 2: Summary of qualitative research***

| **Paper** | **Aim** | **Design** | **Setting** | **Participants** | **Themes related to the perspectives on and experiences of sexuality for older adults** |  |
| --- | --- | --- | --- | --- | --- | --- |
| ([Bauer et al., 2012](#_ENREF_1)) | To explore older adults perceptions on sexual expression in RACFs | Naturalistic inquiry using semi-structured interviews and constant comparative analysis | Six nursing homes in Australia | Older adults in residential aged care (n=16, including 5 with early stage dementia) | Sexual health/activity  Sexuality and expressions of intimacy still matter to older adults  Thinking about previous sexual experience evoked reminiscence or resignation to being 'past it'  Sexuality is a personal matter for older adults  Interaction with healthcare service  RACFs are considered to be unconducive to sexual expression |  |
| ([Clover, 2006](#_ENREF_2)) | To explore how health services can meet the needs of older gay men | In-depth interviews and thematic analysis | Community setting in UK | Gay men (n=not reported) | Interaction with healthcare service  Negative experience in the way homosexuals are treated can prevent them from seeking health care |  |
| ([Colton, 2007](#_ENREF_3)) | To explore how HCPs can promote attainment quality of life through sexual healthcare for older adults | Grounded theory with in-depth interviews and thematic analysis | Community settings in US | 13 males and 12 females, aged 68 to 90 years  Mean age: males 80yrs, females 83yrs  Excluded: long term care residents and people with dementia or wheelchair-bound. | Sexual health/activity  Older adults used specific language to define sexuality and sexual activities, but the majority remain sexual beings and use different ways to express sexuality  Absence of STIs and preventing pregnancy were considered to be the primary issues encompassed by the term sexual health. Interaction with healthcare service  Even if older adults had a sexual problem they rarely raise sexuality in consultation with health providers, due to embarrassment, shame, fear and perceiving sexuality the doctor considers sexuality as not important.  Attitudes to healthcare professionals  Health care provider characteristics influence how open an older adult will be about sexuality. |  |
| ([Frankowski & Clark, 2009](#_ENREF_5)) | To explore older adults' experience of sexuality and intimacy in assisted living facilities | Compilation from two ethnography studies and a mixed methods study | Assisted living facilities in the US (n=13) | 259 interviews with residents, family members and staff members at 13 assisted living facilities | Sexual health/activity  Sexual interactions occur and older adults in assisted living have varying interests in sexuality and intimacy that is often dependent on personal circumstance.  Gossip amongst residents occurs.  Expression of sexual orientation beyond heteronormative presentation is rare in the assisted living setting.  Older adults may use metaphorical sexual language.  Attitudes to healthcare professionals  Staff members are not always respectful regarding sexuality and intimacy. |  |
| ([Gledhill & Schweitzer, 2014](#_ENREF_6)) | To explore the experiences of older adults' with biomedicalisation of sexuality with a focus on use of pharmaceuticals | Phenomenology with purposive sampling, semi-structured interviews, thematic analysis using an interpretive approach. | Community setting in Australia | Older adults (n=8)  Characteristics:   - 24% female - age range 65-84 - all married, divorced or widowed | Interaction with healthcare service  Some older adults do not feel comfortable discussing sexual dysfunction with GPs, and as a result simply put up with loss of function. |  |
| ([O'Brien et al., 2011](#_ENREF_13)) | To describe experience with sexuality in health care settings of patients who have been treated for prostate cancer | Purposive sampling, in-depth interviews and thematic analysis using One Sheet of Paper (OSOP) method | General practice in UK | Men who have been treated for prostate cancer (n=35)  Partners (n=18) were sometimes involved or observing the interview  Characteristics:   - age range 59 to 82 years - 70% aged over 65 years - Range of socioeconomic backgrounds and treatment histories | Interaction with healthcare service  There is limited follow up of psychosexual concerns for older men with prostate cancer.  Older men may conceal their psychosexual problems and consider health professionals to not think them important.  Lack of continuity of care and rapport with staff is a barrier to receiving help with psychosexual problems following prostate cancer.  Health professionals may not address psychosexual issues with older men following prostate cancer due to their age. |  |
| ([Slinkard & Kazer, 2011](#_ENREF_16)) | To explore experiences related to HCPs regarding HIV, STI and sexuality | Focus groups and content analysis | Not stated.  Study in the US. | Two gender-specific focus groups  5 males (mean age 82 yrs)  9 females (mean age 77yr)s | Interaction with healthcare service  Older adults report receiving limited screening or advice from health professionals regarding HIV or STIs. |  |
| ([Tzeng, Lin, Shyr, & Wen, 2009](#_ENREF_18)) | To explore characteristics and contexts related to sexual behaviours among institutionalised older adults with dementia | Grounded theory, interviews and behaviour observation. | Long term care facilities in Taiwan | Male residents with dementia who display sexual behaviours (n=12)   - Mean MMSE 12   Formal caregivers (n=12)   - 83% female - 50% religious - 83% currently married | Sexual health/activity  Different sexual behaviours are displayed by older adults with dementia in long term care.  Predisposing circumstances include having opportunity, a cooperative target, personal space without privacy.  Responses from other residents and caregivers vary (positive, neutral and negative) and may be related to culture. |  |
| ([Hillman, 2008](#_ENREF_8)) | The perspective is from a psychotherapist point of view | Opinion supported by references | N/A | N/A | Sexual health/activity  Older adults may experience body image alteration, fear or complications related to sexual activity as a result of chronic or acute illness.  Older adults may experience alterations to sexual activity as a result of medication side effects. |  |
| ([Lichtenberg, 2014](#_ENREF_10)) | The perspective is from a occupational therapist point of view. | Opinion supported by references | N/A | N/A | Interaction with healthcare service  Older adults may be reserved regarding communication with their primary physician due to fear of dismal or disinterest from the healthcare professional  Older adults prefer the healthcare professional to be the first to raise sexual health discussions |  |
| ([Parker, 2006](#_ENREF_14)) | The paper includes perspective on how older adults view sexuality. | Opinion supported by references | N/A | N/A | Sexual health/activity  The sexual experience of older adults may be influenced by age-related changes including:   - Menopausal changes and erectile dysfunction (ED) - Changes to body shape and appearance - Changes to fitness - Loss of self-esteem and confidence - Illness (e.g. arthritis) - Medications |  |
