# Supplementary material for: Let's talk about sex: older people's views on the recognition of sexuality and sexual health in the health‐care setting
Source: Health Expect. 2015 Oct 8;19(6):1237–50. doi: 10.1111/hex.12418 (PMC6456814; doi:10.1111/hex.12418)
Supplement: Supplementary file 2 — Table S3. Excluded papers. [file HEX-19-1237-s002.docx]

***Table3: Excluded studies***

| **Reasons for exclusion:**  1. Participants not reported as aged > 65 years  2. Paper does not report on older people’s attitudes toward management of sexuality in healthcare settings  3. Systematic or literature review  4. Insufficient relevant/valid research results to report  5. Opinion paper offering no unique information not reported in included research  6. Book reviews, letters to editor, conference abstracts other excluded citation types | |
| --- | --- |
| Aversa, A., Donini, L. M., Bruzziches, R., Lacava, R., Mattace Raso, F., & Sinclair, A. (2014). Coordinating care aspects related to sexual health in the aging male. *International Journal of Endocrinology, 2014*(653587) | 2 |
| Balami, J. S. (2011). Are geriatricians guilty of failure to take a sexual history? *Journal of Clinical Gerontology and Geriatrics, 2*(1), 17-20 | 2 |
| Bar-Chama, N., Snyder, S., & Aledort, L. (2011). Sexual evaluation and treatment of ageing males with haemophilia. *Haemophilia, 17*(6), 875-883 | 1 |
| Barrett, C. M. (2011). Auditing organisational capacity to promote the sexual health of older people. *Journal of Applied Psychology, 7*(1), 31-36 | 2 |
| Bartlik, B. D., Rosenfeld, S., & Beaton, C. (2005). Assessment of sexual functioning: Sexual history taking for health care practitioners. *Epilepsy & Behavior, 7*(2), 15-21 | 1 |
| Bauer, M., McAuliffe, L., Nay, R., & Chenco, C. (2013). Sexuality in Older Adults: Effect of an Education Intervention on Attitudes and Beliefs of Residential Aged Care Staff. *Educational Gerontology, 39*(2), 82-91. | 2 |
| Bauer, M., Nay, R., & Mcauliffe, L. (2009). Catering to Love, Sex and Intimacy in Residential Aged Care: What Information is Provided to Consumers? *Sexuality and Disability, 27*(1), 3-9 | 2 |
| Bauer, M., Nay, R., Tarzia, L., Fetherstonhaugh, D., Wellman, D., & Beattie, E. (2013). 'We need to know what's going on': Views of family members toward the sexual expression of people with dementia in residential aged care. *Dementia* | 2 |
| Beaulaurier, R. L., Craig, S. L., & De La Rosa, M. (2009). Older Latina women and HIV/AIDS: an examination of sexuality and culture as they relate to risk and protective factors. *Journal of Gerontological Social Work, 52*(1), 48-63 | 2 |
| Bentrott, M. D., & Margrett, J. A. (2011). Taking a Person-Centered Approach to Understanding Sexual Expression among Long-term Care Residents: Theoretical Perspectives and Research Challenges. *Ageing International, 36*(3), 401-417 | 2 |
| Béphage, G. (2008 ). Meeting the sexuality needs of older adults in care settings. *Nursing & Residential Care, 10*(9), 448-452 | 3 |
| Bouman, W. P., Arcelus, J., & Benbow, S. M. (2007). Nottingham Study of Sexuality and Ageing (NoSSA II). Attitudes of care staff regarding sexuality and residents: a study in residential and nursing homes. *Sexual & Relationship Therapy, 22*(1), 45-61. | 2 |
| Bradway, C., & Strumpf, N. (2008). Seeking care: women's narratives concerning long-term urinary incontinence. *Urologic Nursing, 28*(2), 123 | 1,2 |
| Burd, I. D., Nevadunsky, N., & Bachmann, G. (2006). Impact of physician gender on sexual history taking in a multispecialty practice. *Journal of Sexual Medicine, 3*(2), 194-200 | 1 |
| Cahill, S., & Valadéz, R. (2013). Growing Older With HIV/AIDS: New Public Health Challenges. *American Journal of Public Health, 103*(3), e7-e15 | 1,3 |
| Chandler, M., Margery, M., Maynard, N., Newsome, M., South, C., Panich, E., & Payne, R. (2004). Sexuality, older people and residential aged care. *Geriaction, 22*(4), 5-11. | 2 |
| Chervenak, J. L. (2010). Reproductive aging, sexuality and symptoms. *Semin Reprod Med., 28*(5), 380-387 | 1,2 |
| Clark-German, T. (2014). *A Study on Internal/External Homophobia and the Impact on Long-Term Health Outcomes: The Effect on Gay, Lesbian, Bisexual, and Transgender Elders in Health Care and Their Decision to Disclose Their Sexual Orientation to Healthcare Providers.* (Doctorate of Medical Humanities), Drew University, Madison, New Jersey | 1 |
| Concannon, L. (2009). Developing Inclusive Health and Social Care Policies for Older LGBT Citizens. *British Journal of Social Work, 39*(3), 403-417 | 1,2 |
| Connolly, M.-T., Breckman, R., Callahan, J., Lacks, M., Ramsey-Klawsnik, H., & Solomon, J. (2012). The sexual revolution's last frontier: How silence about sex undermines health, well-being, and safety in old age. *Generations, 36*(3), 43-52 | 2 |
| Corona, G., Rastrelli, G., Maseroli, E., & Forti, G. (2013). Sexual function of the ageing male. *Best Practice & Research Clinical Endocrinology & Metabolism, 27*(4), 581-601 | 1,2 |
| Cosby, R. (2008). *We Expect To Be Treated The Same": A qualitative study with aging same-sex couples and long-term care.* Open Access Dissertations and Theses. Paper 4752 | 1,2 |
| Crisp, C., Wayland, S., & Gordon, T. (2008). Older gay, lesbian, and bisexual adults: Tools for age-competent and gay affirmative practice. *Journal of Gay & Lesbian Social Services, 20*(1-2), 5-29 | 1,2 |
| Cronin, A., Ward, R., Pugh, S., King, A., & Price, E. (2011 ). Categories and their consequences: Understanding and supporting the caring relationships of older lesbian, gay and bisexual people. . *International Social Work, 54*(3), 421-435 | 1,2 |
| Darnaud, T., Sirvain, S., Igier, V., & Taiton, M. (2013). A study of hidden sexuality in elderly people living in institutions. *Sexologies, In Press*. | 4 |
| Di Napoli, E., Breland, G., & Allen, R. (2013). Staff Knowledge and Perceptions of Sexuality and Dementia of Older Adults in Nursing Homes. *Journal of Aging and Health, 25*(7), 1087-1105. | 2 |
| Dickey, G. (2012). Survey of Homophobia: Views on Sexual Orientation From Certified Nurse Assistants Who Work in Long-Term Care. *Research on Aging, 35*(5), 563-570. | 2 |
| Doll, G. M. (2013). Sexuality in Nursing Homes. *Journal of Gerontological Nursing,, 39*(7), 30-37. | 2 |
| Doll, G. A. (2013). Sexuality and long-term care: do they mix? *Aging Today, 34*(1), 11-15. | 5 |
| Donaldson, W. V. (2014). *Exploring staff clinical knowledge and practice with lgbt residents in long-term care: a grounded theory of cultural competency and training needs.* (Doctor of Philosophy), Colorado State University, Fort Collins, Colorado. | 2 |
| Everett, B. (2008). Supporting sexual activity in long-term care. *Nursing Ethics, 15*(1), 87-96. | 2 |
| Fenge, L., & Hicks, C. (2011). Hidden lives: the importance of recognising the needs and experiences of older lesbians and gay men within healthcare practice. *Diversity in Health & Care, 8*(3), 147-154 | 1 |
| Forte, D., Wells, D., & Cotter, A. (2007). Chapter 10: Intimacy, Sex and Sexuality. In R. Neno (Ed.), *Older People and Mental Health Nursing: A Handbook of Care.* : Blackwell Publishing | 1,2 |
| Fredriksen-Goldsen, K. I., Hoy-Ellis, C. P., Goldsen, J., Emlet, C. A., & Hooyman, N. R. (2014). Creating a Vision for the Future: Key Competencies and Strategies for Culturally Competent Practice With Lesbian, Gay, Bisexual, and Transgender (LGBT) Older Adults in the Health and Human Services. *Journal of Gerontological Social Work, 57*(2-4), 80-107 | 2 |
| Freeman, S., Sousa, S., & Neufeld, E. (2014). Sexuality in Later Life: Examining Beliefs and Perceptions of Undergraduate Students. *Gerontology & Geriatrics Education, 35*(2), 200-213. | 2 |
| Gilmer, M. J., Meyer, A., Davidson, J., & Koziol-McLain, J. (2010). Staff beliefs about sexuality in aged residential care. *Nursing Praxis in New Zealand, 26*(3), 17-24. | 2 |
| Gott, M., Hinchliff, S., & Galena, E. (2004). General practitioner attitudes to discussing sexual health issues with older people. *Social Science & Medicine, 58*(11), 2093-2103. | 2 |
| Hafford-Letchfield, T. (2008). What's love got to do with it? Developing supportive practices for the expression of sexuality, sexual identity and the intimacy needs of older people. *Journal of Care Services Management, 2*(4), 389-405 | 2 |
| Hayward, L. E., Robertson, N., & Knight, C. l. (2013). Inappropriate sexual behaviour and dementia: An exploration of staff experiences. *Dementia, 12*(4), 1-18. | 2 |
| Heath, H. (2011). Older People in Care Homes: Sexuality and Intimate Relationships. *Nursing Older People, 23*(6), 14-20. | 2 |
| Heath, H. (2012). Supporting sexuality and intimate relationships. *Nursing & Residential Care, 14*(9), 475-477. | 2 |
| Helmes, E., & Chapman, J. (2012). Education about sexuality in the elderly by healthcare professionals: a survey from the Southern Hemisphere. *Sex Education, 12*(1), 95. | 2 |
| Heron, J., & Taylor, S. (2009). Nurse manager perceptions regarding sexual intimacy rights of aged care residents: an exploratory Queensland study. *Practice Reflexions, 4*(1), 16-25 | 2 |
| Higgins, A., Barker, P., & Begley, C. M. (2004). Hypersexuality and dementia: dealing with inappropriate sexual expression. *British Journal of Nursing, 13*(22), 1330-1334 | 2 |
| Hillman, J., & Hinrichsen, G. A. (2014). Promoting an affirming, competent practice with older lesbian and gay adults. *Professional Psychology: Research and Practice, 45*(4), 269. | 2 |
| Hillman, J. (2011). A Call for an Integrated Biopsychosocial Model to Address Fundamental Disconnects in an Emergent Field: An Introduction to the Special Issue on "Sexuality and Aging". *Ageing International, 36*(3), 303-312 | 2 |
| Hinchliff, S., Gott, M., & Galena, E. (2004). GPs' perceptions of the gender-related barriers to discussing sexual health in consultations: A qualitative study. *European Journal of General Practice, 10*(2), 56-60 | 1 |
| Hinrichs, K. L., & Vacha-Haase, T. (2010). Staff perceptions of same-gender sexual contacts in long-term care facilities. *Journal of Homosexuality, 57*(6), 776-789 | 2 |
| Horner, B., Mcmanus, A., Comfort, J., Freiiah, R., Lovelock, G., Hunter, M., & Tavener, M. (2012). How prepared is the retirement and residential aged care sector in Western Australia for older non-heterosexual people? *Quality in Primary Care, 20*(4), 263-274 | 2 |
| Hordern, A. J., & Street, A. F. (2007). Constructions of sexuality and intimacy after cancer: patient and health professional perspectives. *Social Science & Medicine, 64*(8), 1704-1718 | 1 |
| Hughes, A. K. (2013). Mid-to-late-life women and sexual health: communication with health care providers. *Family Medicine, 45*(4), 252-256 | 1 |
| Hughes, A. K. (2011). HIV knowledge and attitudes among providers in aging: results from a national survey. *AIDS Patient Care and STDs, 25*(9). | 2 |
| Hughes, M. (2004). Privacy, Sexual Identity and Aged Care. *Australian Journal of Social Issues, 39*(4), 381-392 | 1,2 |
| Hughes, M. (2006). Queer ageing. *Gay and Lesbian Issues and Psychology Review, 2*(2), 54-59 | 2 |
| Hughes, M. (2007). Older Lesbians and Gays Accessing Health and Aged-Care Services. *Australian Social Work, 60*(2), 197-209 | 1 |
| Hughes, M. (2008). Information placed in trust: older gay men and social workers on talking about sexual identity in aged care. *Geriaction, 26*(1), 15-20. | 2 |
| Jackson, N. C., Johnson, M. J., & Roberts, R. (2008). The potential impact of discrimination fears of older gays, lesbians, bisexuals and transgender individuals living in small- to moderate-sized cities on long-term health care. *Journal of Homosexuality, 54*(3), 325-339 | 1 |
| Jacobs, R. J., & Kane, M. N. (2010). HIV-related stigma in midlife and older women. *Social Work in Health Care, 49*(1), 68-89 | 2 |
| Jacobson, S. A. (2013). Sexuality and Long-Term Care: Understanding and Supporting the Needs of Older Adults, by Gayle Appel Doll. *Journal of Gerontological Social Work, 56*(4), 378-380 | 6 |
| Johnson, B. K. (2013). Sexually transmitted infections and older adults. *Journal of Gerontological Nursing, 39*(11), 53-60 | 2 |
| Johnson, B. K. (2004). Sexuality and heart disease: implications for nursing. *Geriatric Nursing, 25*(4), 224-226 | 1 |
| Kaaki, B., & Kingsberg, S. A. (2007). Evaluation and treatment of sexual dysfunction in an older woman. *Journal of Clinical Outcomes Management, 14*(2), 110-120 | 1 |
| Kettl, P. (2008). Inappropriate sexual behavior in long-term care. *Annals of Long Term Care, 16*(12), 29-35 | 2 |
| Knochel, K. A., Croghan, C., F., Moone, R. P., & Quam, J. K. (2012). Training, Geography, and Provision of Aging Services to Lesbian, Gay, Bisexual, and Transgender Older Adults. *Journal of Gerontological Social Work, 55*(5), 426-443 | 2 |
| Knochel, K. A., Quam, J. K., & Croghan, C. F. (2011). Are old lesbian and gay people well served?: Understanding the perceptions, preparation, and experiences of aging services providers *Journal of Applied Gerontology, 30*(3), 370-389 | 2 |
| Langer-Most, O., & Langer, N. (2010). Aging and sexuality: how much do gynecologists know and care? *Journal of Women & Aging, 22*(4), 283-289 | 2 |
| Lee, M. G., & Quam, J. K. (2013). Comparing supports for LGBT aging in rural versus urban areas. *J Gerontol Soc Work, 56*(2), 112-126 | 1,2 |
| Lee, D. M., Tajar, A., Ravindrarajah, R., Pye, S. R., O'Connor, D. B., Corona, G., O'Connell, M., Gielen, E., Boonen, S., Vanderschueren, D., Pendleton, N., Finn, J. D., Bartfai, G., Casanueva, F. F., Forti, G., Giwercman, A., Han, T. S., Huhtaniemi, I. T., Kula, K., Lean, M. E. J., Punab, M., Wu, F. C. W., & O'Neill, T. W. (2013). Frailty and sexual health in older European men. *Journals of Gerontology Series A: Biological Sciences & Medical Sciences, 68*(7), 837-844 | 2 |
| Lekas, H.-M., Schrimshaw, E. W., & Siegel, K. (2005). Pathways to HIV testing among adults aged fifty and older with HIV/AIDS. *AIDS Care, 17*(6), 674-687 | 1,2 |
| Lenahan, P. M., & Ellwood, A. L. (2004). Sexual health and aging. *Clinics in Family Practice., 6*(4), 917-  939 | 2 |
| Lichtenberg, P. A. (2014). Sexuality and Physical Intimacy in Long-Term Care. *Occupational Therapy in Health Care, 28*(1), 42-50 | 2 |
| Lightbody, E., Jackson, G. A., & Lithgow, S. (2013). Sexualtiy, dementia and the care home. *Journal of Dementia Care, 21*(1), 28-31 | 5 |
| Lightbody, E., Jackson, G. A., & Lithgow, S. (2013). Sexualtiy, dementia and the care home, part two. *Journal of Dementia Care, 21*(2), 28-30 | 5 |
| Lochlainn, M. N., & Kenny, R. A. (2013). Sexual activity and aging. *J Am Med Dir Assoc., 14*(8), 565-572 | 1,2 |
| Low, L., Lui, M., Lee, D., Thompson, D., & Chau, J. (2005). Promoting Awareness of Sexuality of Older People in Residential Care. *Electronic Journal of Human Sexuality, 8* | 3 |
| Maes, C. A., & Louis, M. (2011). Nurse Practitioners' Sexual History-Taking Practices with Adults 50 and Older. . *Journal for Nurse Practitioners, 7*(3), 216-222 | 2 |
| Mahan Buttaro, T., Koeniger-Donohue, R., & Hawkins, J. (2014). Sexuality and quality of life in aging: Implications for practice. *Journal for Nurse Practitioners, 10*(7), 480-485 | 2 |
| Mahieu, L., de Casterle, B. D., Van Elssen, K., & Gastmans, C. (2013). Nurses' knowledge and attitudes towards aged sexuality: Validity and internal consistency of the Dutch version of the Aging Sexual Knowledge and Attitudes Scale. *Journal of Advanced Nursing, 69*(11), 2584-2596 | 2 |
| Malatesta, V. J. (2007). Sexual problems, women and aging: an overview. *Journal of Women & Aging, 19*(1-2), 139-154 | 1 |
| McAuliffe, L., Bauer, M., & Nay, R. (2007). Barriers to the expression of sexuality in the older person: the role of the health professional. *International Journal of Older People Nursing, 2*(1), 69-75. | 2 |
| McGrath, M., & Lynch, E. (2014). Occupational therapists' perspectives on addressing sexual concerns of older adults in the context of rehabilitation. *Disability & Rehabilitation, 36*(8), 651-657 | 2 |
| McIntyre, M., & McDonald, C. (2012). The limitations of partial citizenship: health care institutions underpinned with heteronormative ideals. *Advances in Nursing Science, 35*(2), 127-134 | 2 |
| Mellor, R. M., Greenfield, S. M., Dowswell, G., Sheppard, J. P., Quinn, T., & McManus, R. J. (2013). Health care professionals' views on discussing sexual wellbeing with patients who have had a stroke: a qualitative study. *PLoS ONE, 8*(10), e78802 | 2 |
| Moreira, E. D., Brock, G., Glasser, D. B., Nicolosi, A., Laumann, E., Paik, A., Wang, T., & Gingell, C. (2005). Help‐seeking behaviour for sexual problems: the Global Study of Sexual Attitudes and Behaviors. *International Journal of Clinical Practice, 59*(1), 6-16 | 2 |
| Myers, R. (2007). Sexual assault against the elderly presents complex reporting and treatment issues. . *Victimization of the Elderly and Disabled, 10*(1), 6-8 | 2 |
| Nay, R., McAuliffe, L., & Bauer, M. (2007). Sexuality: from stigma, stereotypes and secrecy to coming out, communication and choice. *International Journal of Older People Nursing, 2*(1), 76-80 | 2 |
| Nusbaum, M. R. H. (2005). Sexual health in aging men and women: addressing the physiologic and psychological sexual changes that occur with age. *Geriatrics, 60*(9), 18-23 | 1 |
| Orel, N. A., Stelle, C., Watson, W. K., & Bunner, B. L. (2010). No one is immune: a community education partnership addressing HIV/AIDS and older adults. *Journal of Applied Gerontology, 29*(3), 352-370 | 2 |
| Parker, S. (2006). What barriers to sexual expression are experiences by older people in 24-hour care facilities? *Reviews in Clinical Gerontology, 16*(4), 275-279 | 2 |
| Peate, I. (2004). Sexuality and sexual health promotion for the older person. *British Journal of Nursing, 13*(4), 188-193 | 2 |
| Peate, I. (2013). Caring for older lesbian, gay and bisexual people. *British Journal of Community Nursing, 18*(8), 372 - 374. | 1,2 |
| Penhollow, T. M., Young, M., & Denny, G. (2009). Predictors of quality of life, sexual intercourse, and sexual satisfaction among active older adults. *American Journal of Health Education, 40*(1), 14-22 | 2 |
| Phillips, J., & Marks, G. (2006). Coming out, coming in: How do dominant discourses around aged care facilities take into account the identities and needs of ageing lesbians? *Gay & Lesbian Issues and Psychology Review, 2*(2), 67-77 | 1 |
| Phillips, J., & Marks, G. (2008). Ageing lesbians: Marginalising discourses and social exclusion in the aged care industry. *Journal of Gay & Lesbian Social Services, 20*(1-2), 187-202 | 1 |
| Popeo, D. M., Sewell, D. D., Johnson, K. K., & Abrams, J. M. (2014). Addressing sex and sexuality with older adults: An opportunity to learn and practice effective communication techniques. *American Journal of Geriatric Psychiatry, 1)*, S5-S6 | 6 |
| Porter, K. E., & Krinsky, L. (2014). Do LGBT Aging Trainings Effectuate Positive Change in Mainstream Elder Service Providers? *Journal of Homosexuality, 61*(1), 197-216 | 2 |
| Ports, K. A., Barnack-Tavlaris, J. L., Syme, M. L., Perera, R. A., & Lafata, J. E. (2014). Sexual health discussions with older adult patients during periodic health exams. *Journal of Sexual Medicine, 11*(4), 901-908 | 1 |
| Price, B. (2009). Exploring attitudes towards older people's sexuality. *Nursing Older People, 21*(6), 32-39 | 2 |
| Price, E. (2005). All but invisible: older gay men and lesbians. *Nursing Older People, 17*(4), 16-18. | 2 |
| Raglan, G., Lawrence, H. r., & Schulkin, J. (2014). Obstetrician/gynecologist care considerations: practice changes in disease management with an aging patient population. *Women's Health, 10*(2), 155-160 | 2 |
| Ratner, E. S., Erekson, E. A., Minkin, M. J., & Foran-Tuller, K. A. (2011). Sexual satisfaction in the elderly female population: A special focus on women with gynecologic pathology. *Maturitas, 70*, 210-215 | 2 |
| Reingold, D., & Burros, N. (2004). Sexuality in the nursing home. *Journal of Gerontological Social Work, 43*(2/3), 175-186 | 2 |
| Rheaume, C., & Mitty, E. (2008 ). Sexuality and intimacy in older adults. *Geriatric Nursing, 29*(5), 342-349 | 2 |
| Roach, S. (2004). Sexual behaviour of nursing home residents: staff perceptions and responses. *Journal of Advanced Nursing, 48*(4), 371-379. | 2 |
| Saunamäki, N., Andersson, M., & Engström, M. (2010 ). Discussing sexuality with patients: nurses' attitudes and beliefs. *Journal of Advanced Nursing, 66*(6), 1308-1316 | 2 |
| Skultety, K. M. (2007). Addressing issues of sexuality with older couples. *Generations, 31*(3), 31-37 | 2 |
| Somes, J., & Donatelli, N. S. (2012). Sex and the Older Adult. *Journal of Emergency Nursing, 38*(2), 168-170 | 5 |
| Snyder, R. J., & Zweig, R. A. (2010). Medical and Psychology Students' Knowledge and Attitudes regarding Aging and Sexuality. *Gerontology & Geriatrics Education, 31*(3), 235-255 | 2 |
| Stark, S. W. (2006). HIV After Age 55. *Nursing Clinics of North America., 41*(3), 469-479 | 1,2 |
| Syme, M. L. (2014). The evolving concept of older adult sexual behavior and its benefits. *Generations, 38*(1), 35-41 | 2 |
| Tabak, N., & Shemesh-Kigli, R. (2006). Sexuality and Alzheimer's Disease: Can the Two Go Together? *Nursing Forum, 41*(4), 158-166 | 2,5 |
| Tarzia, L., Bauer, M., Fetherstonhaugh, D., & Nay, R. (2013). Interviewing Older People in Residential Aged Care About Sexuality: Difficulties and Challenges. *Sexuality & Disability, 31*(4), 361-371 | 2 |
| Tarzia, L., Fetherstonhaugh, D., & Bauer, M. (2012). Dementia, sexuality and consent in residential aged care facilities. *Journal of Medical Ethics, 38*(10), 609-613 | 2 |
| Taylor, A., & Gosney, M. A. (2011). Sexuality in older age: essential considerations for healthcare professionals. *Age & Ageing, 40*(5), 538-543 | 1,2 |
| Teitelman, J. (2006). Sexual abuse of older adults: appropriate responses for health and human services providers. *Journal of Health and Human Services Administration, 29*(2), 209-227 | 2 |
| Tolley, C., & Ranzijn, R. (2006). Predictors of heteronormativity in residential aged care facilities. *Australasian Journal on Ageing, 25*(4), 209-214 | 2,4 |
| Tolley, C., & Ranzijn, R. (2006 ). Heteronormativity amongst staff of residential aged care facilities. *Gay and Lesbian Issues and Psychology Review, 2*(2), 78 | 4 |
| Wallace, M., Boltz, M., & Greenberg, S. A. (2008). Sexuality assessment for older adults. *American Journal of Nursing, 108*(7), 57-58 | 1 |
| Wallace, M., & Safer, M. (2009). Hypersexuality among cognitively impaired older adults. *Geriatric Nursing, 30*(4), 230-237 | 2 |
| Wallace, M. A. (2008). Assessment of sexual health in older adults. *American Journal of Nursing, 108*(7), 52-61 | 2 |
| Walsh, K. E., & Berman, J. R. (2004). Sexual dysfunction in the older woman: an overview of the current understanding and management. *Drugs & Aging, 21*(10), 655-675 | 2 |
| Ward, R., Vass, A. A., Aggarwal, N., Garfield, C., & B Cybyk, B. (2005). A kiss is still a kiss? The construction of sexuality in dementia care. *Dementia, 4*(1), 49-72 | 2 |
| Watters, Y., & Boyd, T. V. (2009 ). Sexuality in later life: opportunity for reflections for healthcare providers. *Sexual & Relationship Therapy, 24*(3-4), 307-315 | 2 |
| Wilson, M. M. (2006). Sexually transmitted diseases in older adults. *Current Infectious Disease Reports, 8*(2), 139-147 | 2 |
| Yan, E., Wu, A. M., Ho, P., & Pearson, V. (2011). Older Chinese men and women's experiences and understanding of sexuality. *Culture, Health &Sexuality, 13*(9), 983-999 | 2 |
| Yechezkel, R., & Ayalon, L. (2013 ). Social Workers' Attitudes towards Intimate Partner Abuse in Younger vs. Older Women. *Journal of Family Violence, 28*(4), 381-391 | 2 |
| Yee, L. (2010). Aging and sexuality. *Australian Family Physician, 39*(10), 718-721 | 2 |
